# Correction to: Brain-derived exosomes from dementia with Lewy bodies propagate α-synuclein pathology

**DOI:** 10.1186/s40478-020-01006-4

**Published:** 2020-08-04

**Authors:** Jennifer Ngolab, Ivy Trinh, Edward Rockenstein, Michael Mante, Jazmin Florio, Margarita Trejo, Deborah Masliah, Anthony Adame, Eliezer Masliah, Robert A. Rissman

**Affiliations:** 1 Department of Neurosciences UCSD School of Medicine, La Jolla, CA 92093 USA; 2 Department of Pathology, UCSD School of Medicine, La Jolla, CA 92093 USA; 3 Veterans Affairs San Diego Healthcare System, San Diego, CA 92161 USA

**Correction to: Acta Neuropathol Commun 5, 46 (2017)**

**https://doi.org/10.1186/s40478-017-0445-5**

Following publication of the original article [1], the authors would like to correct the means of anaesthesia prior to euthanasia reported in the Methods section.

- **The sentence currently reads**: Four weeks post injection, mice were anesthetized with chloral hydrate (Sigma, C8383) and transcardially perfused with 0.9% saline.
- **The sentence should read**: Four weeks post injection, mice were anesthetized with an intramuscular injection of a stock anesthetic cocktail solution containing Ketamine (Ketaset from Zoetic Inc., 150 mg/kg) and Xylazine (XylaMed from VetOne, 15 mg/kg), and transcardially perfused with 0.9% saline. Following perfusion, the animals were then decapitated.
